# Molecular analysis confirms the long-distance transport of *Juniperus ashei* pollen

**DOI:** 10.1371/journal.pone.0173465

**Published:** 2017-03-08

**Authors:** Rashmi Prava Mohanty, Mark Alan Buchheim, James Anderson, Estelle Levetin

**Affiliations:** 1 Department of Biological Sciences, The University of Tulsa, Tulsa, Oklahoma, United States of America; 2 Environmental Allergy Assays, London, Ontario, Canada; The Ohio State University, UNITED STATES

## Abstract

Although considered rare, airborne pollen can be deposited far from its place of origin under a confluence of favorable conditions. Temporally anomalous records of Cupressacean pollen collected from January air samples in London, Ontario, Canada have been cited as a new case of long-distance transport. Data on pollination season implicated *Juniperus ashei* (mountain cedar), with populations in central Texas and south central Oklahoma, as the nearest source of the Cupressacean pollen in the Canadian air samples. This finding is of special significance given the allergenicity of mountain cedar pollen. While microscopy is used extensively to identify particles in the air spora, pollen from all members of the Cupressaceae, including *Juniperus*, are morphologically indistinguishable. Consequently, we implemented a molecular approach to characterize *Juniperus* pollen using PCR in order to test the long-distance transport hypothesis. Our PCR results using species-specific primers confirmed that the anomalous Cupressacean pollen collected in Canada was from *J*. *ashei*. Forward trajectory analysis from source areas in Texas and the Arbuckle Mountains in Oklahoma and backward trajectory analysis from the destination area near London, Ontario were completed using models implemented in HYSPLIT4 (Hybrid Single-Particle Lagrangian Integrated Trajectory). Results from these trajectory analyses strongly supported the conclusion that the *J*. *ashei* pollen detected in Canada had its origins in Texas or Oklahoma. The results from the molecular findings are significant as they provide a new method to confirm the long-distance transport of pollen that bears allergenic importance.

## Introduction

Long-distance transport of pollen grains has been reported and discussed in several investigations [1–8]. Campbell et al. [7] reported deposits of jack pine and white spruce pollen near Repulse Bay, Northwest Territories, Canada. Results from backward-trajectory analysis indicated that pollen had been transported from central Quebec to Repulse Bay (ca. 3000 km). Similarly, backward trajectories indicated long-distance transport of large numbers of exotic pollen grains from northeastern North America to southern Greenland [5]. Long-distance transport of *Betula* pollen grains from southwestern Russia, The Baltic states and Poland to Finland were also documented and analyzed by trajectory analysis [3]. Numerous other investigators have also used trajectory analysis to confirm long-distance transport of pollen grains [1, 2, 4, 6, 8]. In each case, a small percentage of released pollen reached the turbulent layer of the atmosphere; this percentage has been described as the “escape fraction” and is the volume of pollen available for long-distance transport [9].

**Fig 1:**
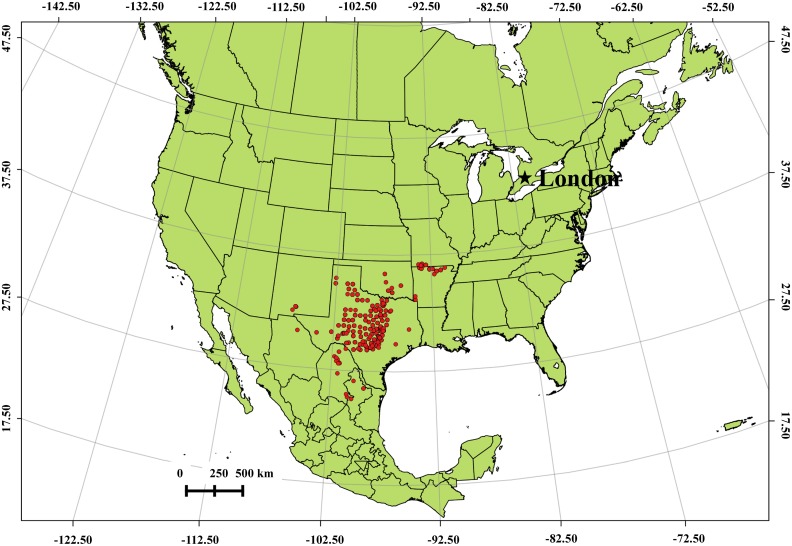
*Juniperus ashei* distribution map in United States.

While most incidents of long-distance transport are regarded as rare events, the transport of *Juniperus ashei* (mountain cedar) pollen into the Tulsa, Oklahoma, USA atmosphere is thought to occur each winter and has been the focus of several investigations [6, 8, 10–12]. Pollen of *J*. *ashei* is highly allergenic and the major cause of hay-fever in Texas [13]. In fact, *J*. *ashei* is the most allergenic species of Cupressaceae in North America and is considered equivalent to ragweed in its allergenic potential [14]. The most extensive *J*. *ashei* populations grow along the limestone slopes of the Edwards Plateau in central Texas [15]. Smaller communities of *J*. *ashei* occur to the south in northern Mexico and to the north in the Arbuckle Mountains of south central Oklahoma and the Ozark Mountains of northwest Arkansas and southeast Missouri (Fig 1) [15, 16]. Pollen release in this species is distinctive in that it occurs in winter, from early December to early February. Unfortunately, *Juniperus* pollen cannot be distinguished morphologically at the species level; in fact, pollen from members of Cupressaceae can only be identified to the family level [17]. Nevertheless, the winter pollination season of *J*. *ashei*, the data from air sampling in the *J*. *ashei* woodlands of Texas and Oklahoma, the prevailing wind patterns, and trajectory analyses have all supported the hypothesis that the Cupressaceae pollen registered in Tulsa during December and January is due to the long-distance transport of *J*. *ashei* pollen from populations in southern Oklahoma and central Texas [8, 12]. Tulsa, in northeast Oklahoma, is approximately 220 km from the *J*. *ashei* population in the Arbuckle Mountains in southern Oklahoma and 800 km from the larger population in central Texas.

Several factors contribute to long-distance transport of *J*. *ashei* pollen. These include small size (ca. 19–22 μm in diameter), light weight (ca. 2.45 x 10^−6^ mg to 4.6 x 10^−6^ mg) [8, 10, 13, 14, 18, 19] and meteorological factors that include strong temperature and pressure gradients [18]. Smaller pollen grains have an advantage over larger pollen grains as they are lighter with less inertia, easier to be removed from microsporangia, more likely to travel long distances and float in the airstream [20]. The weather patterns in the southern Great Plains of the USA are dominated by dry, cold air that moves east out of the Rocky Mountains and south from the Canadian Arctic, which collides with warmer, more humid air of the tropical Atlantic region to the southeast. The collision between these weather patterns results in region-wide atmospheric instability [21] which can capture and carry microscopic particles over long-distances. *Juniperus ashei* pollen concentrations in Tulsa, Oklahoma, correlate with regional southerly winds and with south to north pressure gradients from the mesoscale weather systems that move across the region [8, 10, 14].

Another important factor that may influence transport is moisture. During rain, scales on the microsporangiate cones of conifers close preventing pollen release, and airborne pollen grains are washed from the atmosphere. In contrast, relative humidity of less than 50% enhances pollen release and transport [18]. Changes in relative humidity affect pollen weight, diameter and the settling rate. Water vapor is absorbed through the exine and affects the airborne transport of pollen by changing the weight, size and shape of the pollen grains. Moist air interferes with the cone scale separation and may inhibit pollen dispersal due to hygroscopic weight gain. Increased humidity also leads to changes in settling velocity, which ranges from 0.8 cm/sec to 2.4 cm/sec in case of *Juniperus* pollen [16, 19, 22, 23].

Although pollen from Cupressaceae can be found in the air spora throughout North America, it is noteworthy when detected in Canadian air in January. A previous study used HYSPLIT trajectory analysis to show that temporally anomalous Cupressaceae pollen registered in London, Ontario, Canada on 27 Jan 1999 likely originated from the Edwards Plateau region near Austin, Texas, a distance of approximately 2400 km [18]. The air mass trajectories showed the potential of transport to London, Ontario, but the inability to distinguish *J*. *ashei* pollen by microscopy left questions as to the identity and, therefore, the source. A definitive answer to the question of long-distance (>2000 km) transport of *J*. *ashei* pollen would require the application of some form of molecular characterization.

A growing body of research has demonstrated how molecular evidence can be used to address a host of pollen biology questions. Molecular tools have been developed to characterize single pollen grains [24–31] or pollen in bulk [32]. Molecular characterizations of pollen origin have also been used for forensic analysis of genetically modified organisms [33], in tests for pollen used in honey production [34], in characterizations of pollen pellets [35] and in testing pollinator efficiency [36]. Although the vast majority of research has focused on modern pollen, a molecular tool kit has been developed for the study of ancient pollen [37, 38]. Quantitative PCR methods have been adapted to provide powerful tools for pollen counting [39–42] including recent work in our labs ([43, 44]; see below). The methods described in these investigations allowed researchers to identify and, in some cases, quantify pollen. However, none of these studies used molecular methods to test hypotheses about long-distance transport of pollen registered in air samples.

As noted above, recent work in the Levetin lab has confirmed the utility of PCR (quantitative and traditional) coupled with species-specific primers for the identification of pollen from several species of *Juniperus*, including *J*. *ashei* [43, 44]. Moreover, the results from these molecular analyses confirmed the presence of *J*. *ashei* pollen in Tulsa air samples on 11 days in December 2013 and January 2014 as well as on 9 days in December 2014 and January 2015 [44]. These observations demonstrated that pollen was transported to Tulsa from pollinating populations of *J*. *ashei* well to the south of the city [43, 44]. Could pollen from these same sources be carried in air masses more than 2000 km to the north in Ontario? The objective of this investigation was to apply the PCR methodology to confirm the identity of Cupressacean pollen collected from air samples in London, Ontario during the month of January. Our results identified the anomalous Cupressacean pollen in Canadian air samples as *J*. *ashei*. Thus, these data provide confirmation that aeroallergenic pollen from *J*. *ashei* can be carried for incredibly long distances.

## Materials and methods

### Sample collection

Outdoor air sampling in London, Ontario, Canada (42.98°N, 81.25°W) was conducted by one of the authors (JA) on 15 Jan 2014 using a Buck bio-slide sampler at 2.0 m above ground. The sample was collected in the private parking lot of a building, and owner of the land gave permission for JA to conduct the study at this site. The sampler operated with a flow rate of 15 liters per minute, and the sample was collected for 5 min starting at approximately 11:00 a.m. local time (1600 UTC). The impaction area of the slide was approximately 1 x 14 mm, and the number of Cupressaceae pollen grains was determined by analyzing the entire impaction area at 400x. The slide was subsequently sent to the Levetin Lab for molecular analysis.

### DNA extraction

A sharp razor blade was used to remove the coverslip from the 15 Jan 2014 Canada slide. Any materials attached to the slide and the coverslip were scraped with a razor blade (including the dried pollen stain and mounting medium) and transferred to a 2 ml screw capped tube. Approximately 200–300 μl of Fawley’s extraction buffer [45] was added to the scraped sample, and the tube was placed in 70°C water bath for two hours. This was followed by centrifugation at 16,004 g for 10 minutes to remove the supernatant. One-millimeter glass beads, corresponding to approximately 700 μl of volume, were added to the tube containing the pellet. Lastly, 500 μl of Fawley’s extraction buffer [45] (1 M NaCl, 70 mM Tris, 30 mM Na_2_EDTA, pH 8.6), 15 μl of 10% CTAB extraction buffer and 10 μl of β-mercaptoethanol were added to the tube. The sample was subjected to bead-beating in a mini bead-beater (Biospec Products, Bartlesville, OK USA) for 3 min, followed by incubation at 75°C for one hour. After incubation, 500 μl of chloroform: isoamyl alcohol (49:1) was added, and the tube was centrifuged at 16,004 g for 20 minutes. The aqueous phase was removed and placed in a separate microfuge tube. An additional 500 μl of chloroform: isoamyl alcohol (49:1) was added to the tube with beads and centrifuged. The aqueous phase was added to the previous supernatant and additional 500 μl of chloroform: isoamyl alcohol (49:1) were added and centrifuged at full speed for 20 minutes. A total of 45 μl of 3 M sodium acetate and 900 μl of ice-cold, 100% ethanol were added to the aqueous phase. The tube was gently inverted and kept at -20°C for one hour. The sample was centrifuged at 16,004 g for 15 min to pellet the nucleic acid. The pellet was washed with 70% ethanol and dried by placing it in a 40°C water bath. Special care was taken while removing the supernatant and washing the pellet with 70% ethanol given that the pellet was small. Approximately 20μl of RNase free water was added to the dried pellet.

### PCR analysis

The primers, *asheimat*KF1 and *asheimat*KR1 (Table 1) were designed to amplify the *mat*K region of *J*. *ashei* pollen DNA. Both *in-silico* (comparisons of various sequence reads and/or published Cupressacean data) and experimental analyses were undertaken to assess primer specificity of *asheimat*KF1 and *asheimat*KR1 primers. The specificity experiments were conducted using DNA from *J*. *ashei* (collected from Lampasas, Texas in January 2011), *J*. *virginiana* (collected from Tulsa, Oklahoma in March 2014), *J*. *pinchotii* (Sonora, Texas in October 2013) and *J*. *monosperma* (Santa Fe, New Mexico in 2010) pollen. Primers were also checked with DNA from *J*. *chinensis*, and *J*. *communis* as well as DNA from other Cupressacean genera (*Cryptomeria japonica*, *Thuja* sp. and *Taxodium distichum*). The DNA extracted from the 15 Jan 2014 air sample from London, Ontario, Canada was checked with the above primers for the presence of *J*. *ashei* pollen. A total of 16 μl of PCR mix (final concentration: 10 mM of forward and reverse primer, 5 units of *Taq* polymerase, 10 mM dNTP, 25 mM MgCl_2_, PCR buffer and water; Promega, Madison, WI) was added to the PCR tube. The following amplification protocols were implemented: an initial heating step of 5 min at 94°C, 36 repetitions of each of (1) a denaturation step of 1 min at 94°C, (2) an annealing step of 45s at 54°C and (3) an extension step of 1 min 10s at 72°C. All reactions were terminated following a final extension of 7 min at 72°C. Agarose gel electrophoresis (0.8% in TBE) was performed to verify the presence of suitable amplified products.

**Table 1:** List of species-specific *J*. *ashei* primers used in PCR.

Primer name	Primer sequences (5’-3’)
***asheimat*KF1**	ATCCAACAGGTTATTCTTG
***asheimat*KR1**	TGGATTCTAATGATTTTGT

### HYSPLIT modeling

Air parcel trajectories were produced using PC-windows based registered software HYSPLIT4 (Hybrid Single-Particle Lagrangian Integrated Trajectory). HYSPLIT is a transport and dispersion model, which can compute trajectories for any place on the earth using current or archived meteorological data [46, 47]. The PC-windows version of the model was used to determine forward trajectories of air parcels starting from areas with *J*. *ashei* populations near Austin, Texas (30.4 N, 97.7 W) and the Arbuckle Mountains in Oklahoma (34.5 N, 96.95 W) with source heights 150m, 250m and 500m. These locations were chosen as known source areas of *J*. *ashei* and have been used for previous *J*. *ashei* forecasting from the Levetin lab (13, 20, http://pollen.utulsa.edu/mcforecast.html). Each model run was calculated starting on 12 Jan 2014 at noon (1800 hours UTC) and running for 48 hours. In addition to single origin trajectories from Austin, a trajectory matrix (upper right coordinates were 31.5 N and 97.00 W, lower left coordinates were 29.5 N and 100.5, first grid northeast of lower left point the coordinates are 30.0 W and 100.00 N) was run to produce a grid of trajectories from the *J*. *ashei* population in central Texas with the source height of 500m. Backward trajectories from London, Ontario (42.9 N, 81.2 W) with heights of 2000m, 2250m and 2500m were also produced. To determine all the above trajectories, we used archived meteorological data from the Global Data Assimilation System (GDAS1). For vertical motion, we used input model data which is the default vertical velocity data fields supplied with the meteorological model data.

## Results

**Fig 2:**
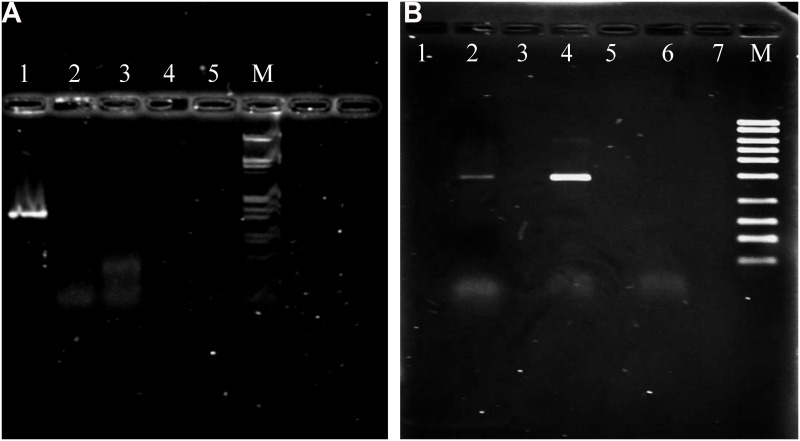
(2A) Agarose gel electrophoresis of PCR products from amplification experiments with *Juniperus* pollen DNA tested with species-specific *J*. *ashei* primers (*asheimat*KF1 and *asheimat*KR1). Lane 1: *Juniperus ashei* pollen DNA, Lane 2: *Juniperus pinchotii* pollen DNA, Lane 3: *Juniperus virginiana* pollen DNA, Lane 4: Blank, Lane 5: Negative control, Lane 6: λ-DNA ladder (*Hin*dIII) as marker (M). (2B) Agarose gel electrophoresis of PCR products obtained from the DNA extracted from the Buck bio-slide sampler from Canada on 15 January 2014 and tested with the primers *asheimat*KF1 and *asheimat*KR1. Lane 1: Blank (No DNA), Lane 2: *Juniperus ashei* pollen DNA obtained from Canada slide on 15 January 2014, Lane 3: Blank (No DNA), Lane 4: Positive control, Lane 5: Negative control (only milliQ water and no DNA), Lane 6–7: Blank (No DNA), Lane 8: *ex*ACTG*ene*^™^ mid- range (300-5000bp) DNA Ladder.

The 15 Jan 2014 London, Ontario slide was analyzed by light microscopy and 12 Cupressaceae pollen grains were identified on the impaction area of the slide. Based on the flow rate and sampling time, the concentration of Cupressaceae pollen at the time of sampling was 160 pollen grains/m^3^. Amplification experiments showed that the primers designed for *J*. *ashei* were species-specific as they failed to amplify pollen DNA from any of the other native *Juniperus* species (*J*. *pinchotii*, *J*. *monosperma*, and *J*. *virginiana;* Fig 2A). Also, the primers failed to amplify DNA from ornamental *Juniperus* species (i.e., *J*. *communis* and *J*. *chinenesis*) or from other ornamental members of the Cupressaceae (i.e., *Cryptomeria japonica*, *Thuja* sp. and *Taxodium distichum*) (data not shown). Also, the *in-silico* analysis undertaken to assess primer specificity of *asheimat*KF1 and *asheimat*KR1 primers confirmed that the *mat*K genes reported for *J*. *horizontalis*, and *J*. *scopulorum* would not be amplified by these primers. In contrast, the DNA for the Cupressacean pollen obtained from the 15 Jan 2014 London, Ontario sample was successfully amplified using primers that were specific for *J*. *ashei* (Fig 2B).

**Fig 3:**
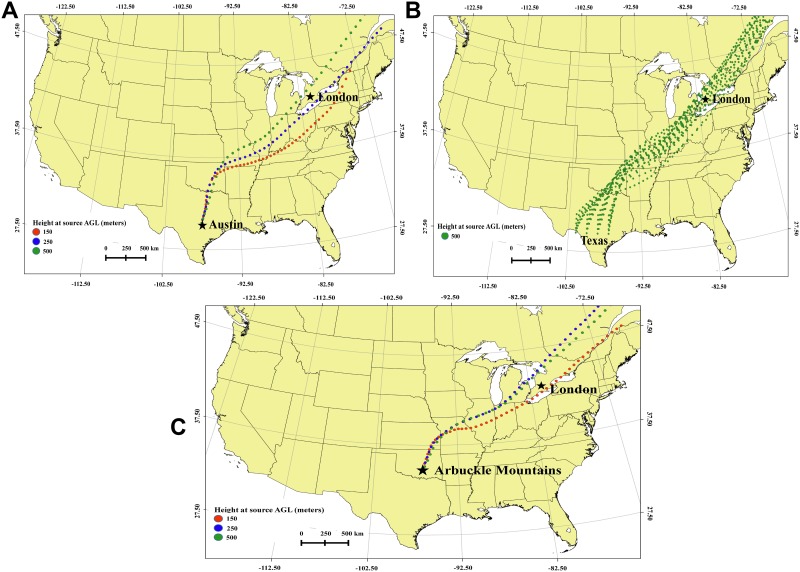
Forward trajectories used in the analysis. (3A) Forward trajectories from Austin, Texas on 12 January 2014 which pass over Tulsa and London, (3B) Forward matrix trajectories from Austin, Texas on 12 January 2014 which pass over Tulsa and London, (3C) Forward trajectories from Arbuckle Mountains, Oklahoma on 12 January 2014, which pass over Tulsa and London.

**Fig 4:**
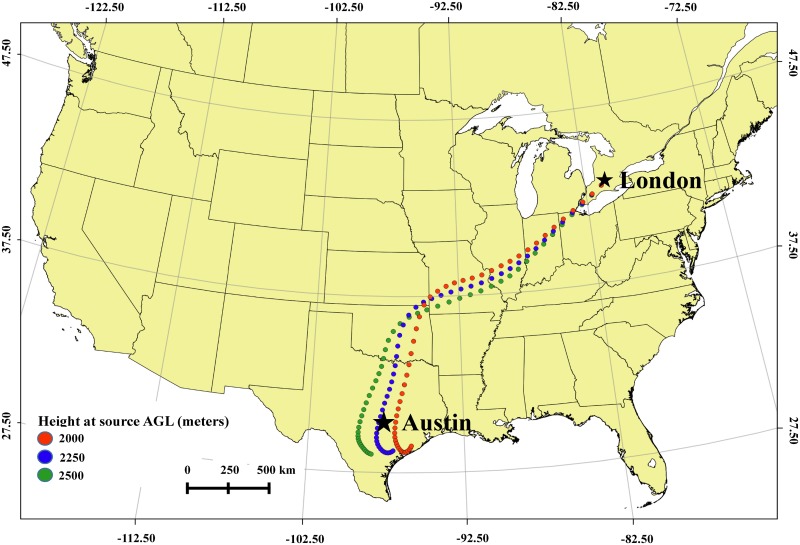
Backward trajectories from London, Ontario, Canada which pass over Arbuckle Mountains, Oklahoma and Austin, Texas.

The HYSPLIT forward trajectories from locations in Texas (Fig 3A and 3B) and the Arbuckle Mountains, in southern Oklahoma (Fig 3C) on 12 Jan 2014 show that the air parcels passed over Tulsa in northeast Oklahoma during 12 Jan and reached London, Ontario, Canada on 14 Jan 2014 at approximately 0000 UTC (Table 2). Backward trajectories from London, Ontario showed the path back to the source areas in Texas and Oklahoma (Fig 4, Table 3).

**Table 2:** Details of forward trajectories from Austin (TX) and the Arbuckle Mountains (OK). Altitudes in bold represent the approximate height and time when the trajectories crossed London, Ontario.

Source Area	Height at Source	FORWARD TRAJECTORIES							
	Date								
	12 January 2014	13 January 2014				14 January 2014			
		Time (UTC)							
	18 hrs	00 hrs	06 hrs	12 hrs	18 hrs	00 hrs	06 hrs	12 hrs	18 hrs
	Meters above ground level								
Austin, TX	150	150	200	750	1000	2000	2250	2750	>3000
	250	250	500	1100	1750	**2000**	**2250**	2750	>3000
	500	500	900	1250	**1750**	**2750**	2900	>3000	>3000
Arbuckle Mts.	150	150	500	1000	1000	**1400**	1500	1900	1600
	250	400	1500	1500	**1500**	1700	1700	1800	.2000
	500	900	2000	1900	**2000**	2000	2500	3000	>3000

**Table 3:** Details of backward trajectories from London (Ontario). Altitudes in bold represent the approximate height and time when the trajectories crossed the Arbuckle Mountains, Oklahoma, and the altitudes in italics represent the approximate height and time when the trajectories crossed Austin, Texas.

Destination Area	Height at Destination	BACKWARD TRAJECTORIES							
	Date								
	14 January 2014	13 January 2014				12 January 2014			
		Time (UTC)							
	00 hrs	18 hrs	12 hrs	06 hrs	00 hrs	18 hrs	12 hrs	06 hrs	00 hrs
	Meters above ground level								
London	2000	1600	1000	600	**250**	***250***	<250	<250	<250
	2250	2150	1400	1100	**250**	***<250***	<250	<250	<250
	2500	2400	1500	1100	**250**	***<250***	<250	<250	<250

## Discussion

Though there are several reports in which long-distance transport of different pollen grains was noted [3, 5, 6], none of these reports used molecular tools to confirm the identity of the pollen. In the previous studies, the inferences were based on a morphological examination of the pollen at the destination site and on the wind trajectory evidence [3, 5, 6]. Our study is the first report in which species-specific primers of *J*. *ashei* were used to amplify pollen DNA (from air samples) to test hypotheses regarding long distance transport. In Canada, four different *Juniperus* species, *J*. *virginiana*, *J*. *communis*, *J*. *horizontalis* and *J*. *scopulorum*, are reported to occur [15]. Pollination for this set of four species begins in mid-March and continues through late April and none of the four are known to produce pollen in either the fall or the winter [15]. Thus, any *Juniperus* pollen found in the London, Ontario air spora during the month of January is likely to be a consequence of long-distance transport. Furthermore, in the unlikely event that the January pollen was from local trees, the only candidate sources are *J*. *virginiana*, *J*. *communis*, *J*. *horizontalis* and *J*. *scopulorum*, and our *J*. *ashei*-specific primers would not amplify DNA from these species. Data from our PCR analyses confirmed that the pollen from the January sample taken in London, Ontario came from *J*. *ashei*. Thus, there is little doubt that the pollen arrived via long-distance transport.

**Fig 5:**
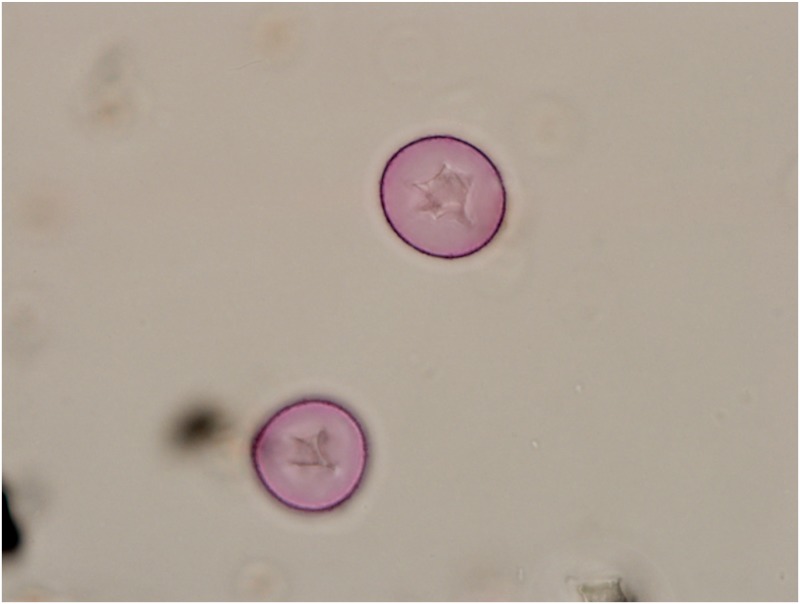
*Juniperus ashei* pollen registered from the Tulsa atmosphere on 12 January 2014 observed under a light microscope.

Moreover, in a separate study [44] air samples from Tulsa, Oklahoma were analyzed by qPCR to evaluate and quantify pollen from various *Juniperus* species. The qPCR data for the 12 Jan 2014 Tulsa sample showed that 55,282 *J*. *ashei* pollen grains were registered in the Tulsa atmosphere on that day; this represents a concentration of 3,839 pollen grains/m^3^ [43, 44] (Fig 5). By contrast, the *J*. *ashei* pollen concentrations in Tulsa were 13 pollen grains/m^3^ on 11 Jan 2014 and 55 pollen grains/m^3^ on 13 Jan 2014 [43, 44]. Thus, the *J*. *ashei* pollen incursion from the south peaked in Tulsa on 12 Jan 2014. Results from the same Tulsa study revealed overlapping pollination days for *J*. *ashei* and *J*. *virginiana* in January. However, the *J*. *virginiana* pollen in the January samples constituted less than 1% of the total Cupressaceae pollen for days analyzed. Similar results also were obtained based on a molecular analysis of archived air samples collected at several sites in Texas; more than 99% of the total Cupressaceae pollen in late January was *J*. *ashei* pollen. By contrast, the archived samples from February contained only *J*. *virginiana* pollen [43]. The *J*. *virginiana* pollination season for Tulsa begins in February and continues through April [10]. In addition, personal observations for over 30 years showed that pollen cones of *J*. *virginiana* in Tulsa generally opened in early to mid-February. Thus, the long-distance transport in mid-January is most likely to be *J*. *ashei* pollen.

Quantitative PCR analysis is especially important when the number and identity of pollen grains is uncertain, and qPCR analysis is the most sensitive molecular technique currently available [42–44]. Since, we already knew that a total of 12 Cupressaceae pollen grains were present on the entire 15 January 2014 air sample slide from Canada, qPCR analysis for this sample was not required. These molecular observations from both Tulsa and London are consistent with an air mass bearing *J*. *ashei* pollen from Texas and southern Oklahoma that carried pollen to Tulsa and eventually reached London, Ontario by 15 Jan 2014.

Our studies have specifically focused on a molecular characterization of pollen from different species of *Juniperus* since morphological analysis is unable to distinguish pollen from any members of the Cupressaceae. Thus, this method of confirming long-distance transport can be implemented for other species where morphological analysis of pollen is unable to clearly distinguish among different species. Our PCR based method is a relatively simple technique to confirm the long distance transport of the pollen grains.

In addition to the molecular evidence, forward trajectories from the HYSPLIT model starting on 12 Jan 2014 from Texas and southern Oklahoma show the *J*. *ashei* pollen grains in the air mass were deposited in Tulsa, Oklahoma on 12 Jan 2014 and in London, Ontario, Canada on 15 Jan 2014. The capture of pollen in London on 15 Jan is consistent with both the trajectories and the settling velocity of *Juniperus* pollen. The trajectories indicate that the air mass with the pollen grains reached London, Ontario at an altitude of 1400 to 2500 meters or higher above ground level; however, the trajectory altitudes only represent the center of the air mass. Pollen grains entrained within the air mass would be at various altitudes above London, Ontario due to diffusion and settling during transport. Based on the altitude of the air mass and the range in settling velocities reported for *Juniperus* pollen, 0.8 cm/sec to 2.4 cm/sec [16, 19, 22, 23], it was estimated that the pollen could reach ground level in 17 to 87 hours. The air sample from London, Ontario was collected on 15 Jan 2014 at 1600 UTC, suggesting the pollen reached ground level in 40 hours. Considering the total travel time of the *Juniperus* pollen from the source and the settling rate of *Juniperus* pollen, a time period of 3 days was required to reach the sampling site in London.

Settling velocities for specific pollen types have frequently shown variability in different studies; in part, this may reflect differences in relative humidity [19, 23, 48]. Studies have found that pollen grains imbibe water as humidity increases, changing the density and the settling rate [19, 23]. The deposition of pollen depends on the settling velocity as well as atmospheric conditions. Although, relative humidity in the upper atmosphere was not determined in this study, the settling time (i.e., 40 hours) to reach the sampler location at 2 meters above ground falls within the range calculated from the different settling velocities (17 to 87 hours) and, therefore, could account for possible atmospheric conditions. Thus, the air-parcel carrying the *J*. *ashei* pollen could have reached the sampling site in London, Ontario, Canada on 15 Jan 2014 at 1600 UTC.

The results of this study using species-specific PCR and trajectory analysis provide the strongest evidence, to date, of long distance transport of *J*. *ashei* pollen from southern Oklahoma and Texas to Canada. These observations suggest that hay fever attributed to the highly allergenic pollen of *J*. *ashei* need not be limited to only those individuals who live in proximity to stands of mountain cedar. We note also that the ability to track pollen over long distances allows scientists to study fundamental questions regarding plant dispersal (e.g., the founder effect by leading edge expansion) [48]. Thus, our results indicate that *Juniperus* would be a good model for testing evolutionary and population genetic hypotheses involving dispersal over long distances.
